# Trends and perspectives in global Food-Based Dietary Guidelines: a narrative review

**DOI:** 10.3389/fpubh.2026.1780915

**Published:** 2026-03-31

**Authors:** Chika Okada, Ryoko Katagiri

**Affiliations:** 1 Center for Nutritional Epidemiology and Policy Research, National Institutes of Biomedical Innovation, Health and Nutrition, Kento Innovation Park, Settsu-shi, Osaka, Japan; 2 Graduate School of Informatics, Chiba University, Chiba-shi, Chiba, Japan

**Keywords:** dietary guideline revisions, Food-Based Dietary Guidelines (FBDGs), national nutrition surveys, nutrition communication, sustainability integration

## Abstract

**Background:**

Food-Based Dietary Guidelines (FBDGs) are key public health tools intended to promote healthy eating and prevent non-communicable diseases. Many countries have recently revised their guidelines, but cross-country comparisons remain limited.

**Objective:**

To compare the latest national FBDGs in selected countries and identify common and divergent approaches in their development and communication.

**Methods:**

A comparative document review was conducted for eight countries with publicly accessible and recently updated FBDGs: the United States, United Kingdom, Australia, Germany, Italy, Denmark, China, and Korea. Official FBDG documents, scientific background reports, and implementation resources were reviewed and compared using a structured narrative approach with attention to governance structure, visual models, food group classification, evidence sources, and sustainability integration. This structured narrative review focuses on selected national FBDGs and does not constitute a comprehensive systematic review of all existing global guidelines.

**Results:**

All eight countries adopted periodic revisions and strengthened alignment with updated nutrition science. Many transitioned from pyramid-style graphics to simplified visual models, with expanded digital engagement and practical consumer resources. Cultural specificity remained visible, such as the Mediterranean pattern in Italy, a plant-forward model in Denmark, and incorporation of fermented foods and rice staples in East Asia. Differences in food grouping and nutrient emphasis reflected national dietary challenges and data availability. Integration of environmental sustainability varied considerably, ranging from explicit inclusion throughout the guidance to minimal or no mention. Public communication strategies increasingly emphasized practicality through consumer testing, targeted messaging, and alignment with food service guidelines.

**Conclusions:**

Contemporary FBDGs show convergence toward improved scientific rigor, cultural appropriateness, and enhanced communication, whereas adopting sustainability occurred at varying levels. Continued evaluation of development processes and public implementation is warranted to strengthen real-world dietary improvement.

## Introduction

Non-communicable diseases (NCDs) remain the leading cause of mortality and morbidity globally, and unhealthy dietary patterns are major contributors to this burden (1–5). According to the Global Burden of Disease Study, dietary risks were responsible for approximately 11 million deaths and more than 250 million disability-adjusted life-years (DALYs) worldwide in 2017, ranking among the leading risk factors, alongside high blood pressure and tobacco use (1). Establishing and promoting healthy eating behaviors is therefore a key strategy for improving population health and extending healthy life expectancy. To support such efforts, Food-Based Dietary Guidelines (FBDGs) have been widely developed and periodically revised by national governments and expert organizations to translate complex nutritional evidence into practical recommendations for the general public (6).

The key messages and visuals of FBDGs are designed to promote adequate nutrient intake and prevent health issues such as lifestyle-related diseases. These components should be grounded in scientific evidence to ensure their credibility and effectiveness (7–11). In addition to scientific validity, the effectiveness of FBDGs depends on their cultural acceptability, feasibility, and clarity. Visual formats, including dietary pyramids and plates, have been increasingly emphasized because they enhance comprehension and facilitate behavioral change by simplifying key nutritional concepts for consumers (12–14). Furthermore, since the publication of the United Nations Sustainable Development Goals and the growing international acknowledgment of the “planetary health” concept, sustainability has become a critical consideration in dietary guidance (15, 16).

In recent years, numerous countries have updated their FBDGs to incorporate emerging evidence on dietary quality, sustainability, and the reduction of ultra-processed foods. Germany released an updated version of its guidelines in 2024 (15, 17), and the United States published its new Dietary Guidelines for Americans in 2026, shortly after our initial data collection period (18, 19). However, previous comparative studies on FBDGs have focused on identifying commonalities and differences in their healthy eating messages based on editions published before 2017 (6) and may not fully capture recent scientific advancements or updates in communication strategies.

To address this gap, the present study investigates recent trends in FBDG revisions, focusing on the scientific evidence, key messages, and visual design approaches adopted by selected countries. It also provides a qualitative study of selected countries, focusing on their unique cultural contexts. By examining these developments, we aim to provide insights that may contribute to the refinement of FBDGs and to future international harmonization in nutrition communication. This analysis further provides guidance for countries updating or creating dietary guidelines, emphasizing the integration of scientific evidence with cultural relevance to enhance effectiveness and public acceptance.

## Methods

Information for this narrative comparative document review was obtained primarily from the official websites of national governments or equivalent public institutions. Between May and July 2024, publicly available data were reviewed to understand the background of FBDG development, theoretical frameworks, scientific foundations, and revision histories. All guidelines were subsequently re-checked for updates through October 2025, and the newly released 2025 U.S. Dietary Guidelines were incorporated based on this final verification. Some U.S. materials, including the MyPlate visual, were previously accessible during our initial review but are no longer available on the official website. Some U.S. materials, including the MyPlate visual and related consumer resources, were accessible during our initial review (May–July 2024) and remained available during the update check conducted through October 2025. However, these materials became unavailable following the release of the updated 2025–2030 Dietary Guidelines in early 2026.

For each country, three types of official documents were included when available: (i) the main FBDG document, (ii) accompanying scientific or background reports, and (iii) implementation resources such as visual tools or consumer-facing materials. These documents were identified through direct navigation of official government websites, using site menus, guideline portals, and internal search functions. We prioritized the most recent and nationally endorsed documents when multiple versions or resources were available. These materials included main dietary guidelines, background reports, and implementation resources. The list of reviewed documents for each country is provided in the References section. From these documents, we extracted standardized information on basic characteristics (guideline name, issuing authority, year of publication, legal basis, and stated objectives), key messages and visualized models (core dietary recommendations and the visual tools employed such as, pyramids, plates, and circular models), and scientific foundations (use of specific algorithms, mathematical models, and national data sources). To ensure consistency, sustainability integration was coded solely based on elements explicitly stated in the official documents. Sustainability was evaluated as explicit inclusion (incorporated into core key messages, food group recommendations, or visual models). Information was collected based on predefined content categories established prior to data collection. The content of each guideline was organized according to common analytical domains to enable cross-country comparison. Data extraction was conducted by an independent reviewer using a standardized template, and the extracted information was subsequently reviewed for consistency by a second researcher. Any discrepancies were resolved through discussion and consensus. Given that many FBDGs have undergone multiple revisions, efforts were made to collect information on revision histories, including the timing, rationale, and major changes over the past two decades. All information was compiled and comparatively analyzed based on official documents, government reports, and publicly available online materials.

We selected eight countries or regions to examine variation in FBDGs across selected nutritional policy environments. Inclusion criteria were as follows: availability of recent and publicly accessible official FBDGs, representation of major high-income economies with global policy influence, coverage of multiple geopolitical regions, and incorporation of distinct and influential dietary traditions. Some countries in other world regions, such as Africa, South America, and the Middle East, have national dietary guidelines, but many of these did not meet our inclusion criteria due to either outdated guidelines or limited available of publicly accessible documentation. Therefore, these regions were not included in this review. The United States (11, 18–37), the United Kingdom (38–41), Germany (42–48), and Italy (49–51) were selected as high-income economies with global policy influence and recently revised FBDGs. Denmark (52–55) was included to represent the Nordic region governed by the Nordic Nutrition Recommendations. China (56–61) and Korea (62–67) were included to represent rapidly evolving dietary transitions in East Asia. Australia (68–72) was added as a nation with a Western dietary context but unique food supply characteristics and dairy consumption patterns. Italy was further emphasized to represent the Mediterranean dietary tradition, which is globally recognized as a model for healthy eating.

To explore how national FBDGs reflect cultural dietary practices, the classification and composition of recommended food groups were examined. This analysis aimed to identify not only shared nutritional principles but also differences rooted in cultural and societal contexts.

## Results

Table 1 summarizes the development, legal basis, and institutional background of national FBDGs in the reviewed countries. In most settings, FBDGs have been established under legal mandates or national strategic frameworks and issued by governmental agencies or authorized professional organizations. Recent revisions indicated a growing emphasis on accommodating diverse dietary practices and incorporating environmental sustainability into public health nutrition policy. In addition to principles derived from nutrient-based reference values, several countries explicitly incorporated traditional eating habits and culturally relevant food practices into their guidance.

**Table 1:** Characteristics of Food-Based Dietary Guideline.

**Country**	**Guideline name**	**Issuing authority**	**Year of publication**	**Legal basis**	**Purpose**	**Target population**	**Visual model (availability)**
United Kingdom	The Eatwell Guide; Government Dietary Recommendations	PHE	2016 (irregular)	Regulations by Public Health England	Promote a balanced and healthy diet	People aged ≥ 2 years across the life course	Present
United States	Dietary Guidelines for Americans	USDA; HHS	2025 (revised every 5 years)	National Nutrition Monitoring and Related Research Act	Promote health and reduce risk of diet-related chronic diseases	All Americans from birth to older adulthood	Present
Australia	Australian Dietary Guidelines	NHMRC	2013 (irregular)	Food and Nutrition Policy (1992)	Promote health and wellbeing; reduce diet-related disease risk	All Australians, except those with specific clinical conditions	Present
Korea	Dietary Guidelines for Koreans	MOHW; MAFRA; MFDS	2021 (revised every 5 years)	Article 14-2, National Nutrition Management Act	Promote healthy dietary behaviors across the life course and reduce nutrition-related disease risk	Entire population across the life course	Present
China	Dietary Guidelines for Chinese Residents	CNS; NHC	2022 (irregular)	National Nutrition Plan 2017–2030	Support healthy living and disease prevention; strengthen health capacity	Healthy individuals aged ≥ 2 years	Present
Germany	Eatwell and Drink Well–DGE Recommendations	DGE	2024 (irregular)	Not specified	Promote health and prevent chronic disease by increasing plant-based foods and reducing animal-based foods; contribute to environmental resource conservation	Healthy adults aged 18–65 years	Present
Italy	Dietary Guidelines for Healthy Eating	CREA	2018 (irregular)	Law No. 258/63; Law No. 70/75; Law No. 454/99	Prevent non-communicable diseases, promote longevity, health, consumer acceptability, and sustainability	General population across the life course	Not specified
Denmark	Official Dietary Guidelines – Healthy and Sustainable; Dietary Guidelines Circle	DFA	2021/2022 (irregular)	Food Act, Chapter 4, Article 10	Indicate healthy and environmentally sustainable food and beverage choices to maintain healthy body weight and reduce disease risk	Healthy individuals aged 2–65 years	Present

PHE, Public Health England; USDA, United States Department of Agriculture; HHS, United States Department of Health and Human Services; NHMRC, National Health and Medical Research Council; MOHW, Ministry of Health and Welfare; MAFRA, Ministry of Agriculture, Food and Rural Affairs; MFDS, Ministry of Food and Drug Safety; CNS, Chinese Nutrition Society; NHC, National Health Commission; DGE, German Nutrition Society; CREA, Council for Agricultural Research and Economics; DFA, Danish Food Administration.

As summarized in Table 2, all eight countries' FBDGs emphasized increased consumption of vegetables, fruits, and whole grains, while recommending reduced intake of sugar, salt, and saturated fat. In contrast, guidance on animal-sourced foods showed substantial cross-country variation. Several countries (e.g., Italy, China, and South Korea) explicitly endorsed a balanced intake of meat and fish, whereas others (e.g., the United Kingdom, Germany, and Denmark) placed stronger emphasis on reduction of red and processed meat. Most guidelines encouraged routine consumption of legumes, although the strength and specificity of these recommendations varied. Water was consistently recommended as the preferred beverage, and many countries advised limiting or avoiding alcohol. Physical activity recommendations were included in most, but not all, guidelines. Notably, the integration of environmental sustainability differed considerably across countries, ranging from explicit inclusion throughout the guidance (e.g., Germany and Denmark) to no explicit mention. Across countries, there was also a growing, but even emphasis on whole or minimally processed foods.

**Table 2:** Comparison of key dietary recommendations in recent Food-Based Dietary Guidelines.

**Category**	**UK**	**USA**	**Australia**	**Korea**	**China**	**Germany**	**Italy**	**Denmark**
Vegetable & Fruit intake	≥ 5 servings/day	Increase; emphasize whole and minimally processed	Actively encourage intake	Encourage intake	Increase intake	At least one serving daily	Actively encourage intake	Plant-based foods at the center; increase fruits and vegetables intake
Meat intake	Limited	Emphasize whole, minimally processed protein foods; limit processed meats	Lean meat as a top protein source	Balanced intake recommended	Reduce intake	Reduce intake	Limit red and processed meats; prefer animal-based foods in moderation	Reduce intake
Fish intake	≥ 2 servings/week; one oily fish	Encourage intake of seafood, particularly omega-3–rich, low-mercury fish	Prefer fish after poultry among animal proteins	Balanced intake recommended	Increase intake	Weekly intake encouraged	Encourage intake of animal foods, including fish	Increase intake
Legumes intake	Encourage beans and pulses	Encourage routine intake	Position legumes after meat proteins	Balanced intake recommended	Increase intake	Regular intake	Actively encourage legumes	Increase intake
Whole-grain intake	Prefer whole-grain products when possible	Increase intake; prefer whole grains	Recommend foods high in whole grains and fiber	Not specified	Increase intake	Choose best available whole-grain option	Actively encourage whole grains	Select whole-grain options
Sugar & salt intake	Reduce intake	Limit added sugars and sodium	Limit intake	Reduce intake	Reduce intake	Restrict intake	Reduce intake	Reduce intake
Fat & oil intake	Consume small amounts	Prefer healthy fats; limit saturated fat	Limit intake	Reduce intake	Reduce intake	Choose plant-based oils	Choose types and reduce amounts	Prefer plant-based, low-fat products
Water intake	6–8 glasses/day	Encourage water over sugary beverages	Drink plenty	Restrict excessive intake	Ensure appropriate hydration	Water is the preferred beverage	Encourage adequate daily hydration	Water is the only recommended beverage
Alcohol intake	No safe level indicated	Recommend avoiding alcohol	Limit intake	Restrict	Reduce	Recommend avoiding	Reduce as much as possible	No safe level indicated
Physical activity	Not included	Recommend regular physical activity	Encourage increased activity	Increase activity levels	Encourage physical activity	Continue being active	Encourage physical activity	Not included
Sustainability/ Environment	Not included	Not included	Not included	Not included	Not included	Explicit inclusion	Not included	Explicit inclusion

The revision history of FBDGs and their visual communication tools is presented in Table 3. All reviewed countries have periodically updated their guidelines to reflect the latest scientific evidence and enhance public comprehension. Over time, visual models have shifted from hierarchical pyramids to more intuitive and consumer-friendly graphics, such as plates and circular models. In the United States, the long-standing MyPlate icon was still in use during our initial review and update check. However, the 2025–2030 Dietary Guidelines for Americans, released in early 2026, introduced a revised visual approach. While the MyPlate icon was no longer featured on the official USDA website at the time of final verification, accompanying materials emphasized a “New Pyramid” graphic and the overarching message “Eat Real Food,” reflecting a shift toward whole, minimally processed foods. Australia has initiated a comprehensive revision process of its 2013 guidelines, scheduled for completion between 2024 and 2026. Recent updates across the selected countries increasingly highlight clarity, practicality, and sustainability, indicating a shared direction toward more accessible communication, although the behavioral impact of these visual or digital tools was beyond the scope of this review.

**Table 3:** Major revisions of national Food-Based Dietary Guidelines and associated visual models.

**Country**	**Visual model/Guideline name**	**Year(s) of major revision**	**Issuing agency/Organization**
United Kingdom	The Balance of Good Health	1994	DH; MAFF; HEA
	The Eatwell Plate	2007	FSA
	The Eatwell Guide	2016	PHE
United States	Dietary Guidelines for Americans (text-based guideline)	Every 5 years (2010, 2015–2020, 2020–2025, 2025-2030)	USDA; HHS
	MyPyramid	2005	USDA; HHS
	MyPlate	2011; 2022 update	
	New Food Pyramid	2025	
Australia	Australian Dietary Guidelines	1992 (policy base); 2003; 2013	NHMRC
Korea	National Food Guides Food Balance Wheel	Major updates linked to DRIs revisions (latest 2020)	KDCA
China	Chinese Dietary Guidelines	2007; 2016; 2022	CNS
Germany	10 Rules of DGE	2017	DGE
	“Gut essen und trinken” DGE Recommendations	2024	
	DGE Nutrition Circle	2005; 2024 update	DGE
Italy	Italian Food-Based Dietary Guidelines	1997; 2003; 2018	INRAN; CREA
Denmark	Official Dietary Guidelines	2009; 2013; 2021	DVFA
	Y-Plate	1980s	
	Official Dietary Guidelines	2022	

DH, Department of Health; MAFF, Ministry of Agriculture, Fisheries and Food; HEA, Health Education Authority; FSA, Food Standards Agency; PHE, Public Health England; USDA, United States Department of Agriculture; HHS, United States Department of Health and Human Services; NHMRC, National Health and Medical Research Council; KDCA, Korea Disease Control and Prevention Agency; CNS, Chinese Nutrition Society; DGE, German Nutrition Society; INRAN, Istituto Nazionale di Ricerca per gli Alimenti e la Nutrizione; CREA, Consiglio per la Ricerca in Agricoltura e l'Analisi dell'Economia Agraria; DVFA, Danish Veterinary and Food Administration.

Although the scientific bases varied by country, all reviewed guidelines were underpinned by on updated evidence, national nutrition survey data, and expert technical input (Table 4). In the United Kingdom, development of the Eatwell Guide incorporated World Health Organization (WHO) recommendations, independent advisory committee reports, consumer research, and environmental impact assessments. In the United States, the 2025–2030 guidelines are grounded in the Scientific Report of the 2025 Dietary Guidelines Advisory Committee and Dietary Reference Intakes, supported by systematic evidence reviews, data analyses, and transparent food pattern modeling procedures. Australia applied a linear programming-based Food Modeling System to ensure feasibility and flexibility in food choices. In East Asia, Korea and China developed their guidance using national dietary surveys and updated dietary reference intakes, with visual tools tailored to reflect national consumption patterns. Korea revised its Food Balance Wheel to six food groups following the 2020 Dietary Reference Intake update and presented visual aids quantifying nutrient contributions per serving using national food composition and Korea National Health and Nutrition Examination Survey. Implementation is linked to municipal health promotion planning. Germany applied mathematical optimization that incorporates environmental impact metrics to inform recommended food proportions in the German Nutrition Circle. Denmark adopted a plant-forward pattern aligned with national food culture and the sustainability-focused Nordic Nutrition Recommendations, with the 2021 guidance currently being evaluated for potential updates following the 2023 Nordic Nutrition Recommendations. Italy emphasized the Mediterranean dietary model supported by WHO and national evidence.

**Table 4:** Scientific foundations for national Food-Based Dietary Guidelines.

**Country**	**Primary scientific sources**	**Methods for deriving food groups and models**	**Implementation and policy integration**
United Kingdom	WHO guidelines; National scientific reports (COMA, SACN, PHE); National Diet and Nutrition Survey	Literature reviews, dietary survey analyses, linear programming, consumer research, and sustainability assessment underplanting the Eatwell Guide	Integrated into national nutrition communication and public health policy
United States	Scientific report of the Dietary Guidelines Advisory Committee; Dietary Reference Intakes	Food Pattern Modeling by age groups; visual tools (e.g., MyPlate and New Pyramid) designed to support guidance rather than independently change behavior	Linked to federal nutrition initiatives, food service guidelines, and related initiatives
Australia	Dietary Reference Values; Food Modeling System	Linear programming to enhance flexibility in food selection and determine recommended servings	Revision process underway (2024–2026)
Korea	Korea National Health and Nutrition Examination Survey; Dietary Reference Intakes for Koreans	Food Balance Wheel with six food groups, revised in alignment with DRI update	Implemented through national and local health promotion plans
China	Scientific Research Report on Dietary Guidelines; Chinese Dietary Reference Intakes	Food Pagoda and simple meal plate visualizations	Promoted through the National Nutrition Plan 2017–2030
Germany	Integrated mathematical optimization model considering nutrition, health, and environment	German Nutrition Circle derived using optimization	Adopted directly into national nutrition policy and institutional food standards
Italy	WHO evidence and national survey data and systematic reviews	Mediterranean diet-based framework	Positioned within obesity prevention and food policy guidance
Denmark	Nordic Nutrition Recommendations; national evidence on sustainability	Circular visual model emphasizing plant-rich diets adapted to Denish context	Used as official guidance incorporation environmental consideration

COMA, Committee on Medical Aspects of Food Policy; SACN, Scientific Advisory Committee on Nutrition; PHE, Public Health England; WHO, World Health Organization.

To compare how national FBDGs reflect distinct food cultures, the classification and composition of recommended food groups in each guideline were examined (Supplementary Table). Across most countries, dietary guidelines commonly recommended the consumption of whole grains and unrefined cereals while limiting refined grains, encouraged the intake of vegetables and fruits, emphasized a balanced intake of animal- and plant-based protein sources, advised reducing added sugar, encouraged limiting oil and improving its quality as preferred fat sources, and highlighted the importance of adequate fluid intake.

## Discussion

This review identified both shared directions and country-specific approaches in the recent development of FBDGs. Across the selected countries, a notable common tendency was the use of more explicit scientific procedures, such as published nutrient-modeling protocols, advisory committee reports, and the incorporation of national dietary survey data. Revisions also prioritized more accessible and behavior-oriented communication through simplified, visually intuitive graphics. Furthermore, sustainability considerations, particularly environmental impact reduction, have increasingly been integrated alongside traditional goals of health promotion and disease prevention. Collectively, these findings indicate that contemporary FBDGs serve not only as evidence-based dietary recommendations but also as culturally grounded public health tools shaped by broader societal priorities.

This review of FBDGs highlights both the commonalities and divergences in dietary guidance across countries. While most guidelines promote increased consumption of fruits and vegetables, whole grains, and reduced intake of sugars and saturated fats, the study also reveals significant variation in how messages are framed, the inclusion of cultural and environmental considerations, and the extent to which food-based recommendations are tailored to local contexts (6). This study compared the classification and composition of food groups recommended in national FBDGs, revealing not only shared nutritional messages but also the ways in which cultural and societal contexts are embedded in these guidelines. Across countries, common messages include promoting the consumption of fruits and vegetables, choosing whole grains, limiting animal fats, and encouraging water intake. However, the emphasis and presentation of these messages vary according to national dietary cultures, public health priorities, and policy objectives. In the United States, the latest guidelines communicate a simplified whole-food–focused message through the new “Eat Real Food” visual, which highlights whole and fresh or minimally processed foods while minimizing highly processed products and added sugars (18, 19). In contrast, Korea's guidelines center on the principle of “eating a variety of foods in balance with emphasizing the intake of vegetables and fruits,” naturally incorporating traditional elements into dietary recommendations (73). Germany's 2024 revised FBDG places sustainability at the forefront, advocating for a plant-based dietary pattern with limited animal-based foods to align health promotion with environmental goals (74). Italy's guidelines emphasize the cultural heritage of the Mediterranean diet, promoting olive oil as the primary fat source and encouraging the consumption of seasonal vegetables (49). These examples illustrate that food group representations in FBDGs function not merely as nutrient delivery frameworks but as culturally resonant messages shaped by national health strategies, food traditions, and educational priority. The findings emphasize the need to align scientific evidence with culturally relevant messaging and policy priorities when developing or revising national dietary guidelines. Moving forward, the formulation of FBDGs will increasingly require the integration of scientific evidence with cultural relevance and community-level applicability to ensure both effectiveness and public engagement.

Key factors to be considered in the development of FBDGs include the relationship between diet and health, the adequacy of nutrient intake, and actual dietary patterns. In recent years, emerging perspectives such as sustainability, the presence of food contaminants, and the potential for personalization have increasingly been incorporated into the conceptual framework of FBDGs (7, 75). As highlighted in the present study, environmental considerations have already been incorporated into the dietary messaging of several countries, with some adopting Food Systems-Based Dietary Guidelines. In particular, Denmark's dietary guidelines aim to foster eating habits that support both human health and environmental sustainability, explicitly emphasizing the principle of “acting in ways that benefit both health and the environment” (52). This reflects a growing expectation that FBDGs can serve as a platform for promoting environmentally sustainable practices. The adoption of national FBDGs has the potential to reduce premature mortality and lower environmental resource demands, including greenhouse gas emissions (76). However, many existing guidelines are not yet fully aligned with global health and environmental targets, underscoring the need for clearer recommendations, particularly regarding the limitation of animal-based foods and the promotion of plant-based alternatives (76). United Nations Nutrition has recently emphasized the importance of a new generation of dietary guidelines that place sustainability at their core (77). These guidelines aim not only to ensure healthy diets for all but also to respond to the growing need for transforming food systems toward greater sustainability. In line with this perspective, the Food and Agriculture Organization (FAO) has also provided a comprehensive framework for developing dietary guidelines that consider not only health but also environmental, social, and economic sustainability (78).

While FBDGs are readily accessible, challenges remain in raising consumer awareness and encouraging their consistent use. Previous research has shown that many individuals are either unaware of the existence of FBDGs or do not fully understand them, and that levels of comprehension and practical use are influenced by factors such as educational attainment, health literacy, and cultural background (9). While visual representations, such as food models, can enhance understanding, they may also lead to misinterpretation if not carefully designed (9). However, improved comprehension does not necessarily translate into improved adherence, and a considerable portion of the population, in high-income and low- and middle-income countries, does not fully follow national dietary guidelines (79). This gap suggests that, in order to enhance the effectiveness of FBDGs as public health tools, it is essential to design them with the consumer perspective in mind. Providing educational support and ensuring cultural relevance may also play a critical role in improving their accessibility and impact.

Visual models serve as graphic representations of dietary guidelines, illustrating what and how much to eat in an accessible format based on consumer research. These models vary widely in design, from the United Kingdom's Eatwell Guide, which presents recommended food proportions in a detailed and informative layout, to the United States' MyPlate and the newly introduced “Eat Real Food” visual (18, 19, 22, 40). MyPlate has long provided a simplified cue for balanced consumption across five food groups and has supported the development of a wide range of educational materials and practical tools available through user-friendly online platforms. The new U.S. visual advances this approach by adopting a new pyramid format that highlights whole and fresh or minimally processed foods while visually minimizing ultra-processed products and added sugars. The design is paired with the clear message “Eat Real Food” and supported by updated resources. Beyond scientific validity, the effectiveness of FBDGs depends on their cultural acceptability, feasibility, and clarity. Visual formats, including dietary pyramids and plates, should be increasingly emphasized because they enhance comprehension and facilitate behavioral change by simplifying key nutritional concepts for consumers.

This study has several limitations. First, the review was limited to a selected set of eight countries with publicly available and recently updated FBDGs, most of high-income setting. As a result, the findings may not fully represent global diversity, particularly in low- and middle-income settings where dietary transitions are also substantial. Second, the comparison was based on guideline documents and official resources rather than on implementation outcomes or population-level dietary changes. This review also did not assess adherence or behavioral impacts of visual or digital tools, which may differ considerably across countries. Third, methodological details underlying guideline development were not always fully disclosed in public sources, which may have led to incomplete characterization of scientific processes in some cases. Finally, guideline revisions are ongoing in multiple countries, and some developments occurred after our initial data collection, including updates to national dietary guidelines in countries such as the United States. Although many countries regularly update their national dietary guidelines, the global burden of non-communicable diseases remains substantial, suggesting that the real-world impact of such guidelines warrants further evaluation in future research. These limitations suggest that future research should expand the scope to additional regions, incorporate evaluation of real-world guideline utilization, and continue monitoring evolving policy frameworks.

## Conclusion

This study examined and compared the scientific foundations, revision processes, and visual representations of national FBDGs in eight selected countries, illustrating how these guidelines function as frameworks for nutrition policy and as tools that reflect cultural and societal contexts. While the findings cannot be generalized globally, the analysis highlights common approaches to evidence use and mathematical modeling, and communication among the included countries. These insights may serve as a useful foundation for future international comparative research on FBDGs and for supporting the development and revision of guidelines in individual countries, with attention to the both scientific validity and cultural relevance.

## Data Availability

The original contributions presented in the study are included in the article/Supplementary material, further inquiries can be directed to the corresponding author.
