# Supplementary material for: Trends and perspectives in global Food-Based Dietary Guidelines: a narrative review
Source: Front Public Health. 2026 Mar 31;14:1780915. doi: 10.3389/fpubh.2026.1780915 (PMC13078381; doi:10.3389/fpubh.2026.1780915)
Supplement: Supplementary file 1 [file Table_1.pdf]

Supplementary Table. Cross-country comparison of food group recommendations in national FBDGs

	**United Kingdom**<br>The Eatwell Guide	**United States**<br>Dietary Guidelines for Americans	**Australia**<br>The Australian dietary guidelines	**China**<br>Chinese Dietary Guidelines	**Korea**<br>Dietary Guidelines for Koreans	**Germany**<br>DGE-Ernährungskreis	**Iitaly**<br>Linee guida per una sana alimentazione	**Denmark**<br>Om De officielle Kostråd
Grains	Eat plenty of starchy carbohydrates including potatoes, bread, rice and pasta.<br>Choose wholegrain varieties.	Focus on Whole Grains<br>All whole-grain foods and products made with whole grains as ingredients.<br>Whole Grains 2-4 servings (1 serving = 1/2 cup cooked oats, brown rice, barley, quinoa, or buckwheat; 1 slice bread; 1 tortilla)	Eat grain(cereal) :(6+ serves/day)<br>A standard serve is (500kJ) or 1 slice (40g) bread, etc<br>Choose wholegrain.	Eat cereal foods: 200-300 g /day<br>Whole grains and beans: including 50-150 g/day	Eat a balanced diet of fresh vegetables and fruits every day, as well as grains, meat, fish, eggs, legumes, milk, and dairy products.	Eat grain : 5 servings /day<br>(1 serving = 60 g)<br>Whole grains : 1/3+ of grain	Replace refined grains with whole grains.<br>(White and refined flours are not poison.)<br>Bread:(175g/day)<br>Pasta,rice,etc:(120g/day)<br>Bread substitutes: (30 g/week)<br>Breakfast cereals: (60 g/week)	Choose whole grain.<br>Rice can contain arsenic, so it is important to vary between different whole grain products.<br>Eat whole grains up to 90 g/day.
Starchy vegetables (e.g., potatoes)	Keep the skins on potatoes.	Eat vegetables throughout the day<br>Vegetables of all types—dark green; red and orange; beans, peas, lentils, and legumes; starchy; and other vegetables, including fresh, frozen, and canned, cooked, or raw vegetables.<br>3 servings (1 serving = 1 cup raw or cooked; 2 cups leafy greens)	Eat vegetable: Plenty, different types and colour. (6+ serves/day)<br>A standard serve is about 75g (100–350 kJ) or 1/2 cup cooked green or orange vegetables (include potatoes), etc	Potatoes: including 50-100 g/day		Eat potatoes: 1 serving /week<br>(1 serving = 250 g)	Potatoes are also a good source of starch and they are not a substitute for vegetables.<br>Potates (400 g/week)	Include potatoes in your meals several times a week.<br>Potates : 100 g/day
Vegetables	Eat fruit and vegetable up to 5 servings/day.<br>1 serving = approx. 80 g (e.g., 3 heaped tablespoons of vegetables or 1 dessert bowl of salad)		Limit intake of pickeld vegetables.	Account for about 1/2 of the weight in a meal.<br>Fresh vegetables: up to 300 g/day<br>Dark vegetables (dark green, red, orange, and purple vegetables) : account for 1/2 of vegetables.		Eat plenty of colourful fruit and vegetables<br>Vegetables and Fruit : 5 servings /day<br>(1 serving = 110 g)	Eat more fresh fruits and vegetables every day limiting the adding of fats and salt.<br>Choose colourful fruits and vegetables, favouring sesonal ones.<br>Fresh vegetables (500 g/day) or Leafy salads (200 g/day)<br>Only occasionally consume processed foods rich in salt (table olives, etc.)	'6 a day' Eat vegetables and fruits : 600 g/day<br>Choose seasonal vegetables, fruits and berries.<br>Vegetables: 1/2+ of your '6 a day'<br>Eat dark green, red and orange vegetables.
Seaweeds	—		—			—	—	Avoid eating hijiki seaweed
Fruits	A serving is about 80 g or serving of dried fruit: 30 g<br>Fruit juice and smoothies: up to 150ml	Eat fruits throughout the day.<br>Fruits of all types, including fresh, frozen, canned, juiced, and dried fruits.<br>2 servings (1 serving = 1 cup raw; 1/2 cup dried)<br>Limit 100% fruit or vegetable juice or dilute with water	Eat fruit: (≥2 serves/day)<br>A standard serve is about 150g (350kJ)) or<br>1 medium apple or 125ml fruit juice (no added sugar, only occasionally), etc	Eat fruit: Fresh fruit 200-350g /day<br>Juice cannot replace fresh fruit.	—	Juice : 2 servings /week<br>(1 serving = 200 g)	Fresh Fruit (450 g/day)<br>or Dried fruit(unsweetened) (90 g/day)<br>Fruit juice cannot replace a portion of fresh fruit.	Fruit juice < 100 ml/day
Protein food	Eat some beans, pulses, fish, eggs, meat, nuts and other proteins.	Prioritize protein foods per meal.<br>Consume a variety of protein foods from animal sources, including eggs, poultry, seafood, and red meat, as well as a variety of plant-sourced protein foods, including beans, peas, lentils, legumes, nuts, seeds, and soy.<br>3-4 servings (1 serving = 3 oz cooked meat, poultry, or seafood; 1 egg; ½ cup beans, peas, or lentils; 1 oz nuts or seeds; 2 tbsp nut or seed butter; 3 oz soy)	Eat lean meats and poultry, fish, eggs, tofu, nuts and seeds, and legumes/beans:(3+ serves/day)<br>A standard serve is (500-600 kJ)	Eat fish, poultry, eggs and lean meat : an average of 120 to 200 g/day.		—	—	Introduce meat-free days and use less meat in your meals.
Red and processed meat products	Limit processed meats.<br>Choose lean meats.<br>Red or processed meats : up to 70 g/day			Livestock and poultry meat :300-500 g/week.<br>Eat less processed meat products.		Eat meat : up to 300 g<br>1-2 servings/week<br>(1 serving = 120 g (beef, pork, poultry))<br>Eat sausage : 2 disc /week<br>1 disc : 30g	Choose lean ones and remove any visible fat before cooking, when choosing meats<br>Red meat: (100g /week)<br>White meat: (200g /week)	Cut down on meat: 350 g of meat /week<br>Limit beef and lamb in particular.<br>Choose primarily meat and meat products up to 10% fat.<br>Limit processed meat as much as possible.
Eggs				Eat eggs without discarding the yolk		Eat 1 egg (60 g) /week	Eat 2-4 eggs (150 g) /week, spread over several days	Eat 3 eggs /week
Fish	Fish 2+ servings (2 x 140g) /week, one of which is oily.			Fish: twice a week or 300-500 g/week.<br>Give priority to fish.		Eat fish : 1-2 servings /week<br>(1 serving = 120 g)	Eat fish and other seafood, (at least 2-3 times a week) choosing local blue fish.<br>Choose small fish that are eaten with the skin.<br>Fish: (300 g/week)<br>Preserved Fish: (50 g/week)	Eat fish: 350 g/ week<br>(Fatty fish : 200 g/week)<br>(Eat fish twice a week as a main course and several times a week as a cold cut.
Legumes (soybeans, soy products, other beans and peas)	Choose beans, peas and lentils instead of meat.			Eat soy products regularly.<br>(*Beans and whole grains : including 50-150 g /day in cereaal foods)		Consume Legumes: 1 serving /week (125 g cooked)<br>For dried pulses, multiply by 1.8 to convert to cooked weight.	Eat more legumes.<br>Fresh legume: (450 g/week) or Dried legume: (150 g /week)	Eat legumes (cooked): 100 g/day (in addition to the '6 a day').
Nuts and seeds				Eat nuts moderately, and its energy should be included in the total energy a day.		Consume nuts : 1 serving /day<br>(1 serving = 25 g)	Introduce small amounts of nuts into your diet.<br>Nuts(60 g/week)	Eat nuts : 30g /day<br>Look for nuts with no more than 0.8 g of salt per 100 g.
Dairy products	Have some dairy or dairy alternatives ; choosing lower fat and lower sugar options.	Consume Dairy<br>Dairy: Whole, reduced-fat, low-fat, or nonfat dairy products, including fluid, dry, or evaporated milk; yogurt; and cheeses. Lactose-free and lactose-reduced products, as well as fortified dairy alternatives, are also options.<br>3 servings (1 serving = 1 cup milk; 3/4 cup yogurt; 1 oz cheese)	Include Dairy and/or alternatives mostly reduced fat.<br><br>Dairy: (2.5+ serves/day)<br>(1 standard serve = 500–600 kJ)	Choose a variety of dairy products.<br>Dairy : equivalent of 300 ml of liquid milk /day		Eat milk and dairy products : 2 servings /day<br>(1 serving = yogurt 150 g; cheese 30 g; milk 250 g)	Drink a cup of milk or yogurt every day, preferably choosing semi-skimmed milk.<br>Choose leaner cheeses.<br>Milk (375 ml/day) or Yogurt (375 g/day)<br>Cheeses up tp 25 %fat (300 g/week) or Cheeses 25+ %fat (150 g/week)	Go for the Keyhole when choosing dairy products.<br>Choose primarily<br>Skimmed, mini-buttermilk<br>Fermented milk products, such as plain yogurt up to 1.5 %fat<br>Cheeses up to 17 %fat<br>Limit dairy products with a high fat content, such as cream.<br>Milk: 250-350 ml/day<br>Cheeses: about 20 g (1 slice)/day
Sugars and confectionery	Check the label and avoid foods which are high in fat, salt and sugar.<br>Free sugars : up to 5% of the energy<br>Free sugars : up to 30 g/day	Limit added sugars<br>Choose water (still or sparkling) and unsweetened beverages.	Limit intake of foods and drinks containing added sugars.	Limit added sugar up tp 50g /day, (best to control up to 25g/day)<br>Do not drink or drink less sugary drinks.	Less sweet	Limit sweet foods better.<br>Sugar-sweetened beverages are not to be recommended.	Reduce intake of sugar.<br>Sweet baked goods: (60-100 g/week)<br>Limit your consumption of high-sugar products.	Especially limit soda, sweets, cakes, chips and the like.<br>Limit snacks and sweets up to 5 handfuls /week. Don't stock up on snacks and sweets<br>Sweet drink up to 500 ml/week
Seasonings	Try replacing salt with pepper, herbs and spices.<br>Salt : up to 6 g/day	Sodium : <2,300 mg/day<br>Limit highly processed foods	Do not add salt to foods in cooking or at the table.	Eliminate the need to add excessive salt or other condiments to enhance the flavor.<br>Salt : up to 5 g/day	Less salty<br>Choose salt-preserved food less, and prepare foods with less salt.<br>Keep salt intake less than 10 g/day. (Target: gradually reduce to 6 g/day)	A lot of salt also ends up in food when preparing meals at home or adding salt at the table.<br>Salt : <6 g/day	Reduce intake of salt and choose iodine-fortified products.	—
Fats and oils	Saturated fat (men :up to 30 g/day; women: up to 20 g/day)	Incorporate healthy fats<br>4 1/2 servings (1 serving = 1 tsp olive oil or butter)	Replace saturated fats with polyunsaturated and monounsaturated fats.	Develop a light diet and eat less fried foods.<br>Cooking oil :25 -30 g/day<br>Trans fatty acids :<2 g/day	Less fatty meats and fried foods	Limit fatty foods better.<br>Choose vegetable oils.<br>Vegetable oils: 1 tbsp/day<br>Butter and margarine: 1 tbsp/day (1 tbsp = 10 g)	Prefer vegetable-based seasoning fats, especially extra virgin olive oil.<br>olive oil (30 ml/day) or butter, fats of animal origin (30 g/day)	Choose vegetable oils over hard fats when cooking.
Alcoholic beverages	Limit alcohol: up to 14 units /week.<br>(1 unit = 10 ml or 8 g of pure alcohol)	Limit Alcoholic Beverages	Limit alcohol: up to 2 standard drinks/day<br>(up to 4 standard drinks on a single occasion)<br>A serve of Discretionary choices provides: 200ml wine (2 standerd drinks), etc	Limit alcohol: <15g/day	Moderate alcohol	Avoid alcoholic beverages.	The least possible.<br>Avoidance of alcohol from any source.<br>Limit the quantities (women and elderly: up to 1 alcoholic unit/day (e.g., a glass of wine); men: up to 2 alcoholic units/day)	Limit alcohol.<br>Alcohol up to 10 units/week, up to 4 units on the same day.<br>(1 unit = 1 regular beer (33 cl., 4.6% vol.); 1 glass of wine (12.5 cl., 12% vol.) ,etc)
Tea, coffee, and water	Drink 6-8 cups/glasses of fluid/day.	Prioritize water (still or sparkling) and unsweetened beverages.	Drink plenty of water.	Drink plenty of water, in small amounts and multiple times.<br>Recommended intake of water : 1.7 L/day for men; 1.5 L/day for women (in mild climates, low physical activity).	Drink plenty of water	Drink water or other caloriefree beverages.	Drink abundant water every day.<br>Water must be the preferred fluid for rehydration.<br>Drinks 8+ glasses (1.6 L) of water /day	Quench your thirst with water.<br>Fruid : 1-1.5 L/day (water, coffee, tea, milk, juice and other beverages)<br>Coffee up to 4 cups/day
Others (high-sodium processed foods)	Check the label and choosing foods that are lower in salt.	Limit Added Sugars, & Refined Carbohydrates	Read labels to choose lower sodium options among similar foods.	To control salt intake, the best way is to buy less high-salt (sodium) foods and eat less preserved foods.		Limit salty foods better. We consume about two-thirds of our daily salt intake through processed foods	Read the labels on packaged products, both artisanal and industrial.	Reduce your salt intake by choosing foods with the Keyhole.

Rows represent food categories; columns represent countries; cells represent recommendation as stated in the original guideline.
Symbol: '—' = Not explicitly mentioned in the guideline.
Units are retained as originally reported (g/day, g/week, servings/day, cups).
Where serving definitions differ, direct quantitative comparability is limited.
Estimated g/day conversions (where feasible) are provided in the 'Estimated_g_per_day' sheet.
tbsp, tablespoon.
Dotted lines indicate subgroups within the same section, with additional explanations inside the dotted area.
Solid lines represent independent groups.
When multiple calorie patterns are provided, values correspond to the 2,000 kcal pattern.
